# Uptake of Nutri-Score during the first year of implementation in Belgium

**DOI:** 10.1186/s13690-020-00492-1

**Published:** 2020-10-23

**Authors:** Stefanie Vandevijvere

**Affiliations:** Sciensano, Service of lifestyle and chronic diseases, J Wytsmanstraat 14, 1050 Brussels, Belgium

**Keywords:** Nutri-Score, Front-of-pack labeling, Supermarkets, Belgium

## Abstract

**Background:**

Front-of-pack (FOP) nutrition labeling has been recommended by the World Health Organization (WHO) as one of a suite of measures needed to improve population diets. The voluntary Nutri-Score FOP labeling system, which was first implemented in France, was approved for implementation in Belgium by the Minister of Public Health in August 2018 and has been officially adopted in Belgium since April 1st 2019. We assessed the uptake of Nutri-Score by food retailers and manufacturers during the first year of implementation in Belgium.

**Main body:**

In November–December 2019, pictures for 1781 products displaying Nutri-Score on the FOP were collected from the five biggest retailers, representing about 10% of products on the market in Belgium. About 90% of products displaying Nutri-Score on the FOP in 2019 were own-brand products from two major food retailers, while the few remainder were branded products. About 56% of products displayed Nutri-Score A or B while 26% of products displayed Nutri-Score D or E.

**Conclusion:**

During the first year of implementation, > 1700 food products displayed Nutri-Score on the FOP in Belgium. While the majority of those products were healthier foods (Nutri-Score A&B), about one quarter of less healthy products (Nutri-Score D&E) already displayed Nutri-Score as well. In the meantime, all five biggest retailers, including discounters, have committed to display Nutri-Score on the FOP from 2020 onwards, which may increase pressure on other food manufacturers to improve uptake of Nutri-Score for branded products.

## Background

Front-of-pack (FOP) nutrition labeling has been repeatedly recommended by the World Health Organization (WHO) as one of a suite of measures needed to improve population diets [1, 2]. The policy objectives of FOP are generally twofold: (i) to provide interpretive information to consumers to inform healthier food choices; and (ii) to encourage the food industry to reformulate their products towards healthier options. The voluntary Nutri-Score FOP labeling system, which was first implemented in France, was approved for implementation in Belgium by the Minister of Public Health in August 2018 and has been officially adopted in Belgium since April 1st 2019. Nutri-Score is calculated based on the energy, saturated fat, total sugar, sodium, and fruit, vegetable, nut and legume contents and, in some instances, the protein and fibre content. Nutri-Score rates the nutritional quality of packaged foods with five colours/letters from red (least healthy) to green (most healthy) [3]. A previous study among 1007 Belgian consumers found that Nutri-Score was the most effective FOP to inform consumers about the nutritional quality of food products, compared to other existing FOP nutrition labels [4].

In November–December 2019 we collected pictures of food products displaying Nutri-Score on the FOP from the five biggest retailers in Belgium to verify uptake of Nutri-Score during the first year of implementation, as well as to assess the types of products that are displaying Nutri-Score.

## Uptake of Nutri-score in Belgium in the first year of implementation

In total 1781 food products with Nutri-Score on the FOP were collected from the stores. This represents roughly 10% of products on the Belgian market. For 50 products (2.8%) pictures taken were of bad quality, leaving 1731 food products to be analyzed. Out of those food products, 33.1% (*N* = 573) displayed Nutri-Score A, 22.8% (*N* = 394) Nutri-Score B, 17.8% (*N* = 308) Nutri-Score C, 17.3% (*N* = 299) Nutri-Score D and 9.1% (*N* = 157) displayed Nutri-Score E (Table 1). The types of food products most frequently carrying Nutri-Score were fruit and vegetable products (18.8%), ready meals, including pizzas (13.4%), dairy products and alternatives (13.3%), bread and bakery products (10.0%) and meat and meat products (8.4%). The types of food products most frequently displaying Nutri-Score A were fruit and vegetable products (*N* = 252, 77%) and grains (*N* = 67, 100%), and the types of food products most frequently displaying Nutri-Score E were bread and bakery products (*N* = 59, 34%) and meat and meat products (*N* = 37, 26%) (Table 1). About 89% of food products displaying Nutri-Score were own-brand products from two major retailers, while the few remainder were branded products from a handful of food manufacturers.

**Table 1:** Uptake of Nutri-Score during the first year of implementation in Belgium (November–December 2019)

	Products		Nutri-Score				
Food category	N	%	A	B	C	D	E
Fruit and vegetable products	326	18,8	252	40	30	4	0
Ready meals, including pizza	232	13,4	52	112	56	12	0
Dairy products and alternatives	231	13,3	56	83	35	55	2
Bread and bakery products	173	10,0	17	26	23	48	59
Meat and meat products	145	8,4	1	29	30	48	37
Fish and fish products	104	6,0	18	33	28	24	1
Cereal and grain products	71	4,1	39	6	21	5	0
Soups and sauces	68	3,9	11	21	12	17	7
Grains	67	3,9	67	0	0	0	0
Meat alternatives	66	3,8	28	17	16	5	0
Spreads and dips	53	3,1	18	9	12	12	2
Chocolate and confectionary	39	2,3	0	0	7	4	28
Beverages	37	2,1	2	3	21	3	8
Ice-cream	31	1,8	3	5	3	16	4
Miscellaneous	28	1,6	9	8	6	5	0
Desserts	20	1,2	0	0	2	18	0
Fats and oils	17	1,0	0	0	0	8	9
Sugar and jam	15	0,9	0	0	4	11	0
Crisps and snacks	8	0,5	0	2	2	4	0
Total	**1731**	**100,0**	**573**	**394**	**308**	**299**	**157**
% of total			**33,1**	**22,8**	**17,8**	**17,3**	**9,1**

In the meantime, one other major retailer and two discounters also committed to display Nutri-Score on the FOP of their food products from 2020 onwards. According to Euromonitor 2018, the three biggest retailers in Belgium also represent the top three packaged food manufacturers in terms of market share, together representing 23.3% of the market share in Belgium. Some retailers are also taking additional actions such as displaying Nutri-Score on shelf labels in store or displaying Nutri-Score on food products online, not only for their own brand products but including all products in store. The high uptake of Nutri-Score by the biggest retailers in Belgium and these additional actions may increase pressure on other food manufacturers to improve uptake of Nutri-Score for branded products, which is currently very limited in Belgium as only a handful of food manufacturers committed to implement Nutri-Score. Recently in May 2020, the European Commission, as part of its farm to fork strategy, announced it will propose harmonized mandatory front-of-pack nutrition labelling taking into account impacts on the single market [5]. This may also further increase pressure on non-retailer food manufacturers to implement Nutri-Score.

Compared to the Health Star Ratings (HSR), a similar voluntary scheme implemented since 2014 in Australia and New Zealand, the uptake of Nutri-Score in Belgium seems to be faster due to strong commitment from the retailers. In New Zealand In 2016, 2 years after adoption of the HSR, 5.3% of packaged food and beverage products surveyed (*n* = 807/15,357) displayed HSR labels [6]. In Australia, 5 years after adoption of the HSR, it can be found on 40.7% of food products [7]. Similarly as in our study, where about three-quarters of products displaying Nutri-Score were A, B or C, in Australia more than three quarters (76.4%) of products displaying HSR had a HSR ≥ 3.0. Products displaying a HSR logo had a significantly higher mean HSR (3.4), compared to products not displaying a HSR logo (2.6) (*p* < 0.001) [7].

## Conclusions

In the first year of implementation in Belgium, Nutri-Score appeared on > 1700 products, roughly 10% of the total food supply. The large majority of these products (~ 90%) were own brand products from two major retailers. While the majority of products displaying Nutri-Score were healthier (A&B), about one quarter of products displayed Nutri-Score D or E on the FOP. All the five biggest retailers in Belgium have now committed to put Nutri-Score on the FOP while the number of packaged food and beverage manufacturers committing remains very low, but may increase due to pressure from the retailers. The latter also shows the necessity of mandatory FOP labeling as per the EU farm to fork strategy to get full uptake of FOP nutrition labeling  across different food and Nutri-Score categories.

## Data Availability

The data are available from the author upon reasonable request.

## Abbreviations

FOP: Front-of-pack
HSR: Health Star Ratings
WHO: World Health Organization

## References

[1] World Health Organization (2013). Global action plan for the prevention and control of noncommunicable diseases 2013–2020.

[2] Kelly B, Jewell J (2018). What is the evidence on the policy specifications, development processes and effectiveness of existing front-of-pack food labelling policies in the WHO European region?.

[3] Chantal J, Hercberg S, Europe WHORO for (2017). Development of a new front-of-pack nutrition label in France: the five-colour Nutri-score. Public Health Panorama.

[4] Vandevijvere S, Vermote M, Egnell M, Galan P, Talati Z, Pettigrew S (2020). Consumers’ food choices, understanding and perceptions in response to different front-of-pack nutrition labelling systems in Belgium: results from an online experimental study. Arch Public Health.

[5] European Commission. A farm to fork strategy for a fair, healthy and environmentally-friendly food system [internet]. Brussels; 2020. Available from: https://ec.europa.eu/food/farm2fork_en.

[6] Mhurchu CN, Eyles H, Choi Y-H. Effects of a voluntary front-of-pack nutrition Labelling system on packaged food reformulation: the health star rating system in New Zealand. Nutrients. 2017;9(8):918. 10.3390/nu9080918 PMC5579711 28829380

[7] Shahid M, Neal B, Jones A. Uptake of Australia’s health star rating system 2014-2019. Nutrients. 2020;12(6):1791. 10.3390/nu12061791 PMC7353262 32560224

